# Cancer-Specific Mortality in Sarcomatoid Renal Cell Carcinoma: A Histological Subtype-Controlled Analysis

**DOI:** 10.3390/jcm15062133

**Published:** 2026-03-11

**Authors:** Michele Nicolazzini, Calogero Catanzaro, Federico Polverino, Michele Petix, Maximilian Filzmayer, Leonardo Quarta, Jordan A. Goyal, Riccardo Schiavina, Nicola Longo, Gennaro Musi, Felix K. H. Chun, Alberto Briganti, Shahrokh F. Shariat, Carlotta Palumbo, Fred Saad, Alessandro Volpe, Pierre I. Karakiewicz

**Affiliations:** 1 Cancer Prognostics and Health Outcomes Unit, Division of Urology, University of Montréal Health Center, Montréal, QC H2X 0A9, Canada; calogero.catanzaro@studio.unibo.it (C.C.); fedepolve97@gmail.com (F.P.); michele.petix@unimi.it (M.P.); filzmayer@med.uni-frankfurt.de (M.F.); quarta.leonardo@hsr.it (L.Q.); jordan.goyal@umontreal.ca (J.A.G.); fred.saad@umontreal.ca (F.S.); pierre.karakiewicz@umontreal.ca (P.I.K.); 2 Division of Urology, Department of Translational Medicine, Maggiore della Carità Hospital, University of Eastern Piedmont, 28100 Novara, Italy; carlotta.palumbo@uniupo.it (C.P.); alessandro.volpe@med.uniupo.it (A.V.); 3 Division of Urology, Department of Oncology, University of Turin, 10043 Orbasano, Italy; 4 Division of Urology, IRCCS Azienda Ospedaliero-Universitaria di Bologna, 40138 Bologna, Italy; riccardo.schiavina3@unibo.it; 5 Department of Neurosciences, Science of Reproduction and Odontostomatology, University of Naples Federico II, 80131 Naples, Italy; nicola.longo@unina.it; 6 Department of Urology, IEO European Institute of Oncology, IRCCS, Via Ripamonti 435, 20141 Milan, Italy; 7 Università degli Studi di Milano, 20122 Milan, Italy; gennaro.musi@ieo.it; 8 Department of Urology, University Hospital, Goethe University Frankfurt, 60596 Frankfurt am Main, Germany; felix.chun@ukffm.de; 9 Division of Experimental Oncology, Unit of Urology, URI–Urological Research Institute, IRCCS San Raffaele Scientific Institute, 20132 Milan, Italy; briganti.alberto@hsr.it; 10 Vita-Salute San Raffaele University, 20132 Milan, Italy; 11 Department of Urology, Comprehensive Cancer Center, Medical University of Vienna, 1090 Vienna, Austria; shahrokh.shariat@meduniwien.ac.at; 12 Department of Urology, Weill Cornell Medical College, New York, NY 10065, USA; 13 Department of Urology, University of Texas Southwestern Medical Center, Dallas, TX 75390, USA; 14 Hourani Center for Applied Scientific Research, Al-Ahliyya Amman University, Amman 19328, Jordan

**Keywords:** renal cancer, histological subtype, sarcomatoid, SEER database, survival

## Abstract

**Introduction:** Sarcomatoid dedifferentiation may be identified in both clear cell renal cell carcinoma (ccRCC) and non-clear cell RCC (nccRCC). Within the SEER database (2010–2021), we tested the effect of sarcomatoid dedifferentiation in first ccRCC and subsequently in nccRCC on cancer-specific mortality (CSM). **Methods:** Separate propensity score matching (PSM) and multivariable competing risks regression (CRR) analyses were first applied to ccRCC with vs. without sarcomatoid dedifferentiation and subsequently to nccRCC with vs. without sarcomatoid dedifferentiation. **Results:** Sarcomatoid dedifferentiation was present in 2496 (3.0%) of 82,146 ccRCC patients and in 501 (1.9%) of 26,584 nccRCC. In ccRCC, after 1:2 PSM, 2496 (100%) patients with sarcomatoid dedifferentiation vs. 4992 (6.2%) patients without sarcomatoid dedifferentiation were included. At 60 months, CSM was 45.7% vs. 33.6% in ccRCC patients with vs. without sarcomatoid dedifferentiation. In CRR sarcomatoid dedifferentiation independently predicted 1.6-fold higher CSM (HR 1.6, *p* < 0.001). In nccRCC, after 1:2 PSM 501 (100%) patients with sarcomatoid dedifferentiation vs. 1002 (3.8%) patients without sarcomatoid dedifferentiation were included. At 60 months, CSM was 41.7% vs. 28.1% in nccRCC patients with vs. without sarcomatoid dedifferentiation. In CRR sarcomatoid dedifferentiation independently predicted 2.0-fold higher CSM (HR 2.0, *p* < 0.001). **Conclusion:** Sarcomatoid dedifferentiation is invariably associated with higher CSM in both ccRCC and nccRCC. However, the detrimental effect of sarcomatoid dedifferentiation in CSM is more pronounced in nccRCC than in ccRCC.

## 1. Introduction

Sarcomatoid dedifferentiation (SD) represents an established adverse prognostic feature that may be present in all histological subtypes of renal cell carcinoma (RCC), which include clear cell, papillary, chromophobe and even rare entities, such as collecting duct RCC [1,2]. Previous large-scale databases analyses have confirmed the adverse prognostic effect of SD in RCC [3,4,5]. However, these analyses either focused only on ccRCC or considered RCC with SD as a homogeneous group, regardless the underlying histological subtype. As such, the detrimental effect on survival of SD on different RCC histological subtypes has not been well explored yet. Indeed, only small-sized, historical studies have attempted to quantify its effect on cancer-specific mortality (CSM) according to histological subtype [6,7,8,9]. These reports often revealed conflicting results. Specifically, three studies, which compared the effect of SD according to its coexistence with clear cell (ccRCC) vs. non-clear cell RCC (nccRCC) histological subtypes, failed to identify a survival disadvantage between RCC histological subtype in patients with SD [6,7,8]. Conversely, in a fourth study, which compared nccRCC with SD to ccRCC with SD, the presence of SD associated with nccRCC exerted a 2.3-fold higher CSM than when SD was associated with ccRCC histological subtype [9]. Consequently, no previous study has examined the effect of SD presence on CSM rates in a controlled fashion. Specifically, it has not been previously quantified to what extent the presence of SD increases CSM relative to its absence in either ccRCC or nccRCC histological subtypes.

We address this specific knowledge gap. Specifically, within the Surveillance, Epidemiology and End Results (SEER) database, we attempted to quantify the potential increase in CSM when SD coexists with ccRCC and, subsequently, we repeated the analysis addressing the potential CSM increase when SD coexists with nccRCC. We hypothesized that SD in ccRCC and nccRCC will be associated with higher CSM. We postulated that the detrimental effect of SD may be more pronounced in nccRCC than in ccRCC.

## 2. Materials and Methods

### 2.1. Study Population

Within the SEER database (2010–2022), we identified patients with histologically confirmed RCC (International Classification of Disease for Oncology [ICD-O] site code C64.9). Within those, individuals with a ccRCC histological subtype (ICD-O-3 codes 8310 and 8005/3) were identified and stratified according to the presence of SD (CS-specific factor 4 codes and Sarcomatoid Features Recode (2010+) codes, Supplementary Table S1). Specifically, ccRCC patients with SD represented the cases, and the remaining ccRCC without SD represented the controls. Subsequently, the same methodology was applied to the patients with the nccRCC histological subtypes (papillary RCC: ICD-O-3 codes 8050 and 8260; chromophobe: ICD-O-3 codes 8270 and 8317; collecting duct: ICD-O-3 codes 8319; and other), where nccRCC patients with SD represented the cases and the remaining nccRCC patients without SD represented the controls. Patients with an unknown histological subtype, unknown TNM stage, and unknown tumor size were excluded, in addition to autopsy-only or death-certificate only observation.

Both cases (with SD) and controls (without SD) were subsequently stratified according to stage (localized—T1-2 N0 M0—vs. regional—T3-4 N0 M0—or Tany—N1 M0—vs. metastatic—Tany Nany M1). Patients with localized and regional stages that were not treated with nephrectomy were not included in the analyses. All analyses and, subsequently, all results were reported and interpreted separately for ccRCC and, subsequently, for nccRCC.

### 2.2. Statistical Analyses

First, patient and tumor characteristics were tabulated according to the presence or absence of SD in ccRCC patients. Subsequently, the tabulation was repeated according to the presence or absence of SD in nccRCC patients. Second, the estimated annual percentage changes (EAPC) for the proportion SD in, first, ccRCC patients and, subsequently, in nccRCC patients were calculated using log-linear regression. Third, two separate one-to-two propensity score matching (PSM) protocols, according to the nearest neighbor method, were applied. The first PSM was applied between ccRCC with SD and ccRCC without SD patients. Subsequently, PSM was applied between nccRCC with SD and nccRCC without SD patients. The PSM relied on patient age (years, continuously coded), sex, race/ethnicity (Caucasian vs. African-American vs. Hispanic vs. Asian/Pacific Islander vs. other), year of diagnosis, tumor size (cm, continually coded), T-stage, N-stage, M-stage and local treatment type. In nccRCC patients, the PSM also relied on histological subtype, namely papillary, chromophobe, collecting duct and other. The aim of the PSM was to eliminate the differences between patients with and without SD that may represent bias or confounders. A one-to-two ratio was selected, as it was the highest possible to maximize the controls while maintaining an absolute standardized mean difference < 0.1 for all variables included in the matching. This approach has been previously used in other population-based analyses [10,11,12,13]. Since the presence of SD defines a high nuclear grade, patients with low-grade ccRCC and papillary RCC were excluded from the matching and from further analyses comparing patients with vs. without SD. Fourth, cumulative incidence plots depicted five-year CSM differences first between ccRCC with vs. without SD and, subsequently, between nccRCC with vs. without SD. Fifth, multivariable competing risks regression (CRR) models addressing CSM were first fitted to test the effect of SD in ccRCC. Subsequently separate CRR models were fitted to test the effect of SD in nccRCC. The covariates were age, sex, race/ethnicity, year of diagnosis, tumor size, stage and local treatment type. In the CRR model fitted on nccRCC patients, the adjusting covariates also included the histological subtype. Finally, subgroups analyses were first applied to ccRCC patients, where the effect of SD was assessed separately in patients with localized, regional, and metastatic stages. Subsequently, separate subgroup analyses were applied to nccRCC in localized, regional, and metastatic stages. Within each stage-specific subgroup, separate PSM was applied and separate multivariable CRR models were fitted. The R software was used for statistical computing and graphics (R version 4.4.1 (14 June 2024); The R Foundation for Statistical Computing, Vienna, Austria). All tests were two-sided with the significance level set at *p* < 0.05.

## 3. Results

### 3.1. Study Population and Temporal Trends

**Figure 1:**
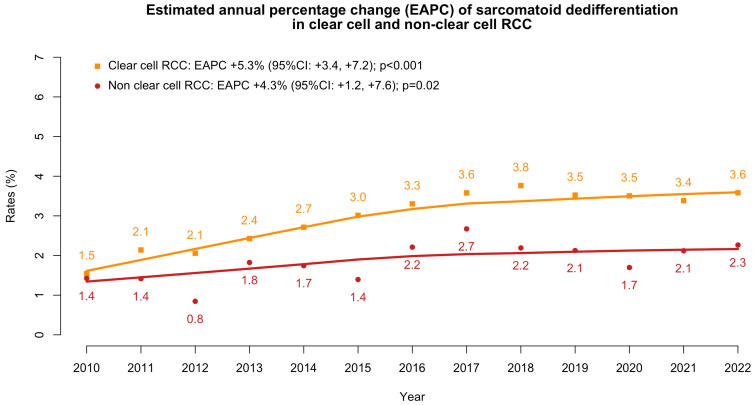
Estimated annual percentage changes (EAPC) in the rates of the presence of sarcomatoid dedifferentiation in clear cell renal cell carcinoma (RCC) and in non-clear cell RCC within the SEER database (2010–2022).

Within SEER (2010–2022), we identified 2977 RCC patients with SD. Of them, 2496 (83.4%) harbored a ccRCC and 501 (16.8%) harbored an nccRCC. Among RCC patients without SD, 79,650 harbored a ccRCC and 26,083 harbored an nccRCC. During the study’s span, the rate of SD significantly increased in both ccRCC patients (from 1.5% in 2010 to 3.6% in 2022; EAPC +5.3%, *p* < 0.001) and nccRCC patients (from 1.4% in 2010 to 2.3% in 2022; EAPC: +4.3%, *p* = 0.02, Figure 1).

### 3.2. Presence vs. Absence of Sarcomatoid Dedifferentiation in Clear Cell RCC

#### 3.2.1. Descriptive Characteristic and PSM

In ccRCC patients, relative to those without SD, patients with SD were older (median age 64 vs. 62 years), more frequently male (69.6% vs. 63.2%), exhibited larger tumor size (median 8.5 vs. 4.2 cm), more frequently harbored either regional (42.7% vs. 21.0%) or metastatic stage (35.5% vs. 7.7%) and were more frequently treated with radical nephrectomy (88.0% vs. 52.8%, all *p* < 0.001, Table 1).

After 1:2 PSM 2496 (100%) ccRCC with SD patients were compared to 4992 (6.2%) ccRCC patients without SD. After PSM no significant residual differences in baseline characteristics were recorded between ccRCC with and without SD (Table 1).

#### 3.2.2. Survival Analyses

**Figure 2:**
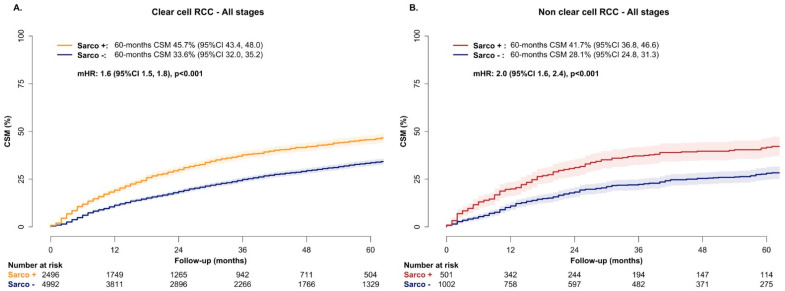
Cumulative incidence plot depicting cancer-specific mortality (CSM) and multivariable competing risk regression predicting CSM in RCC patients with vs. without sarcomatoid dedifferentiation according to histological subtype: (**A**) clear cell RCC; (**B**) non-clear cell RCC. Shaded areas around the curves represent 95% confidence interval. Multivariable CRR was adjusted for age, sex, race/ethnicity, tumor size, stage, local treatment, and year of diagnosis. In the CRR model addressing non-clear cell RCC, histological subtype was also included among adjusted variables.

In cumulative incidence plots, the 60-month CSM rates were 45.7% vs. 33.6% in the ccRCC patients with vs. without SD, respectively (Figure 2A). In the multivariable CRR models addressing CSM after adjusting for OCM and covariates, SD independently predicted higher CSM in ccRCC (HR: 1.6, 95% CI: 1.5–1.8, *p* < 0.001, Figure 2A).

#### 3.2.3. Stage-Specific Subgroup Analyses

**Figure 3:**
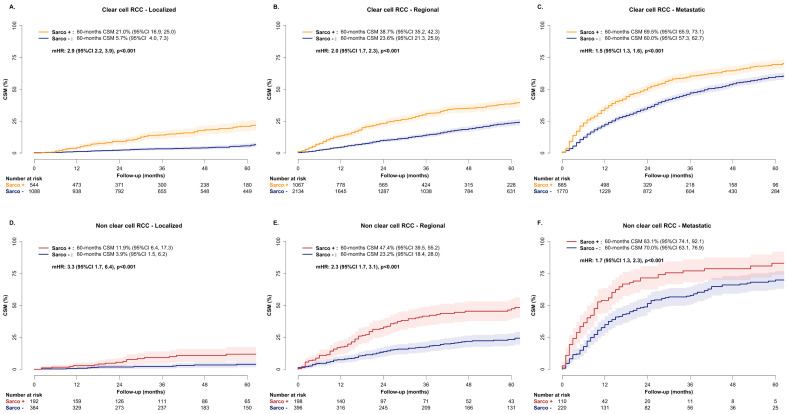
Cumulative incidence plot depicting cancer-specific mortality (CSM) and multivariable competing risk regression predicting CSM in RCC patients with vs. without sarcomatoid dedifferentiation according to histological subtype and stage: (**A**) clear cell RCC, localized stage; (**B**) clear cell RCC, regional stage; (**C**) clear cell RCC, metastatic stage; (**D**) non-clear cell RCC, localized stage; (**E**) non-clear cell RCC, regional stage; (**F**) non-clear cell RCC, metastatic stage. Shaded areas around the curves represent 95% confidence interval. Multivariable CRR was adjusted for age, sex, race/ethnicity, tumor size, stage, local treatment and year of diagnosis. In the CRR model addressing non-clear cell RCC, histological subtype was also included among adjusted variables.

In localized stage ccRCC patients, after 1:2 PSM, multivariable CRR models addressed CSM in 544 (100%) patients with SD vs. 1088 (8.0%) patients without SD. Here, SD independently predicted higher CSM (HR: 2.9, 95% CI: 2.2–3.9, *p* < 0.001, Figure 3A).

In the regional-stage ccRCC patients, after 1:2 PSM, the multivariable CRR models addressed CSM in 1067 (100%) patients with SD vs. 2134 (24.5%) patients without SD. Here, SD independently predicted higher CSM (HR: 2.0, 95% CI: 1.7–2.3, *p* < 0.001, Figure 3B).

In the metastatic-stage ccRCC patients, after 1:2 PSM, the multivariable CRR models addressed CSM in 885 (100%) patients with SD vs. 1770 (66.34%) patients without SD. Here, SD independently predicted higher CSM (HR: 1.5, 95% CI: 1.3–1.6, *p* < 0.001, Figure 3C).

### 3.3. Presence vs. Absence of Sarcomatoid Dedifferentiation in Non-Clear Cell RCC

#### 3.3.1. Descriptive Characteristic and PSM

In nccRCC patients, relative to those without SD, patients with SD exhibited larger tumor size (median 8.0 vs. 3.8 cm), more frequently harbored either regional (39.5% vs. 13.7%) or metastatic stage (22.0% vs. 2.9%) and were more frequently treated with radical nephrectomy (82.2% vs. 43.5%, all *p* < 0.001, Table 2).

After 1:2 PSM 501 (100%) nccRCC patients with SD were compared to 1002 (3.8%) nccRCC patients without SD. After PSM no significant residual differences in baseline characteristics were recorded between nccRCC patients with and without SD (Table 2).

#### 3.3.2. Survival Analyses

In cumulative incidence plots, the 60-month CSM rates were 41.7% vs. 28.1% in nccRCC patients with vs. without SD, respectively (Figure 2B). In multivariable CRR models addressing CSM after adjusting for OCM and covariates, SD independently predicted higher CSM in nccRCC (HR: 2.0, 95% CI: 1.6–2.4, *p* < 0.001, Figure 2B).

#### 3.3.3. Stage-Specific Subgroup Analyses

In the localized-stage nccRCC patients, after 1:2 PSM, multivariable CRR models addressed CSM in 192 (100%) patients with SD vs. 384 (3.1%) patients without SD. Here, SD independently predicted higher CSM (HR: 3.3, 95% CI: 1.7–6.4, *p* < 0.001, Figure 3D).

In the regional-stage nccRCC patients, after 1:2 PSM, multivariable CRR models addressed CSM in 198 (100%) patients with SD vs. 396 (15.2%) patients without SD. Here, SD independently predicted higher CSM (HR: 2.3, 95% CI: 1.7–3.1, *p* < 0.001, Figure 3E).

In the metastatic-stage nccRCC patients, after 1:2 PSM, multivariable CRR models addressed CSM in 110 (100%) patients with SD vs. 220 (51.5%) patients without SD. Here, SD independently predicted higher CSM (HR: 1.7, 95% CI: 1.3–2.3, *p* < 0.001, Figure 3F).

## 4. Discussion

It is generally agreed that presence of SD represents an adverse prognostic factor in both ccRCC and nccRCC. However, robust data validating this statement are not available. Specifically, no study quantified the effect of SD on CSM in a controlled fashion relative to patients without SD in either ccRCC or nccRCC. We addressed this knowledge gap. First, we focused on the effect of SD in ccRCC patients. Subsequently we reassessed this relationship in nccRCC patients. We made several noteworthy observations.

First, SD is rare in ccRCC and even rarer in nccRCC patients. Specifically, the rate of SD was 3.0% in ccRCC and 1.9% in nccRCC. These rates are comparable to those reported by Wang et al. according to the National Cancer Database (NCDB), where SD was recorded in 4890 of 162,773 ccRCC patients (3.0%) and in 1225 of 60,097 nccRCC patients (2.0%) [3]. In Wang et al.’s analysis, moreover, the commonest nccRCC histological subtypes, namely, papillary RCC and chromophobe RCC, were associated with a lower risk of presenting with SD at pathology (OR 0.72–0.74), whereas rarer histological subtypes, such as medullary RCC and collecting duct RCC, were associated with a higher risk of SD presence at pathology [3]. However, due to the smaller sample of nccRCC patients in the current SEER-based study than in the NCDB-based analyses by Wang et al., further stratification of nccRCC histological subtypes was not attempted. Taken together, the current study, as well as that by Wang et al., indicate a higher prevalence of SD in ccRCC than nccRCC histological subtypes.

Second, the current study also provides temporal trends of SD in first ccRCC and, subsequently, in nccRCC. Over the study’s span, an increase in prevalence was recorded in ccRCC (EAPC +5.3%) and, to a lesser extent, in nccRCC (EAPC 4.3%). These observations cannot be compared with previous reports, since none addressed the temporal trend of SD’s prevalence in either ccRCC or nccRCC patients. Although this increase in SD prevalence may reflect a more precise and mindful pathological reporting, rather than a true biological phenomenon, these findings highlight the growing relevance of SD in the RCC landscape and, consequently, support the rationale for the present study.

Third, important differences in baseline characteristics distinguished patients with SD from their counterparts without SD. These differences applied to both the ccRCC and nccRCC histological subtypes. Specifically, patients with SD with both the ccRCC and nccRCC histological subtypes harbored larger tumors (8.5 and 4.2 cm in ccRCC and 8.0 vs. 3.8 cm in nccRCC), with higher rates of regional (42.7% vs. 21.0% in ccRCC and 39.5% vs. 13.7% in nccRCC) and metastatic stages (35.5% vs. 7.7% in ccRCC and 22.0% vs. 2.9% in nccRCC, all *p* < 0.001) than their counterparts without SD. These differences are not unexpected, since previous reports have identified tumor size, nodal involvement and AJCC stage as predictors of SD at diagnosis [3]. However, the current study is the first to separately contrast the characteristics of patients with SD relative to patients without SD within ccRCC and, subsequently, nccRCC patients. This is important, since the absolute difference in stage presentation was different between patients with nccRCC and ccRCC. Specifically, the magnitude of the regional stage increase was more pronounced in nccRCC patients (∆ + 25.8%) than in ccRCC (∆ + 21.7%). Conversely, the magnitude of the metastatic stage increase was more pronounced in ccRCC patients (∆ + 27.8%) than in nccRCC (∆ + 19.1%). These differences in stage distribution between RCC with and without SD underscore the importance of rigorous adjustment, including PSM and multivariable modeling, when evaluating the independent effect of SD on CSM.

Fourth, we tested the effect of SD in ccRCC and identified a 1.6-fold higher CSM rate when SD was present (60-month CSM 45.7% vs. 34.1% in ccRCC patients with vs. without SD, HR 1.6, *p* < 0.001). The same analyses in nccRCC revealed a 2.0-fold higher CSM rate when SD was present (60-month CSM 41.7% vs. 28.1% in nccRCC patients with vs. without SD, HR 2.0, *p* < 0.001). Moreover, in the subgroup analyses that was stratified according to stage (localized vs. regional vs. metastatic), the magnitude of the adverse prognostic effect of SD was invariably stronger in the nccRCC histological subtypes relative to the ccRCC histological subtype, across all stages (HR 3.3 vs. 2.9 in the localized stage; 2.3 vs. 2.0 in the regional stage; 1.7 vs. 1.5 in the metastatic stage), even though the effect of SD in both ccRCC and nccRCC patients progressively decreased from the localized to metastatic stages. These observations validate the adverse prognostic effect of SD in both ccRCC and nccRCC and underline the stronger adverse prognostic effect of SD in nccRCC relative to ccRCC, irrespective of clinical presentation and across all stages. The decrease in the magnitude of the detrimental effect on CSM in advanced stages has already been reported in previous analyses that did not separately address ccRCC and nccRCC [5,14] and may reflect the inherent more aggressive behavior of RCC with SD, which remains more pronounced when compared with a more indolent reference group, such as localized RCC without SD. It is of note that localized RCC with SD is much more prone to recur after treatment than its counterpart without SD, partly explaining the more pronounced detrimental effect on CSM than metastatic RCC [1,15].

The stronger adverse impact of SD on CSM in nccRCC relative to ccRCC observed in the present study may be explained, at least in part, by underlying biological differences. It is notable that cc- and ncc-RCC with SD, though sharing some mutational characteristics, such as RELN mutation, harbor specific genomic alterations according to the underlying histological subtype [1,16]. Specifically, PTEN was associated with ccRCC with SD, NF2 mutations were more frequent in papillary RCC with SD, and TP53 mutations were elevated in chromophobe RCC with SD [1,16]. These findings suggest that the biological heterogeneity of SD may contribute to its variable prognostic impact across RCC subtypes.

Taken together, this study provides the first large-scale, controlled comparison of the prognostic impact of SD within ccRCC and nccRCC separately. Although SD is rare, its prevalence is increasing, and its adverse effect on CSM is consistently more pronounced in nccRCC than in ccRCC across all stages. Therefore, the presence of SD should be carefully taken into account during clinical decision-making in both ccRCC and to a greater extent in nccRCC patients, in whom the current findings indicate a markedly adverse prognostic impact. In these patients treatment and follow-up intensification may be justified and warrants further investigation. Unfortunately, SEER does not capture granular systemic therapy data nor data on follow-up and recurrence, limiting the possibility to explore the potential benefit of such intensifications. On the other hand, SD rarity renders studies focused on patients with SD difficult, especially when SD is associated with nccRCC histological subtypes. To date, indeed, adjuvant immunotherapy has been tested only in ccRCC patients with SD [17]. Similarly, among the novel combination therapy for metastatic RCC, only one was tested in nccRCC metastatic patients with SD [18]. In consequence, the current results help to identify a high-risk subgroup of patients—nccRCC with SD—that warrants specific consideration in future trial design, or at least focused large-scale retrospective analyses to evaluate possible advantages of treatment and follow-up intensification.

Despite the strength of our findings, limitations need to be acknowledged. First, its retrospective nature and the lack of a central revision within the SEER database may have introduced bias and confounders, even though these limitations are shared with previous population-based analyses [3,10,11,14,19]. However, they may be overcome by the advantages of a large-scale database, particularly when studying rare entities such as sarcomatoid RCC. Second, detailed data on the percentage of sarcomatoid features, previously identified as prognostic factors in sarcomatoid RCC [20,21,22], were not available. Third, detailed data on tumor burden, especially in metastatic patients, were not available, as well as disease progression and subsequent treatment. Finally, specific data on patient’s comorbidities and treatment modalities are not available in the SEER, preventing the assessment of the effect of these variables on CSM.

## Figures and Tables

**Table 1:** Descriptive characteristics of clear cell RCC patients with vs. without sarcomatoid dedifferentiation (SD), before and after 1:2 propensity score matching (PSM), relied on age, sex, race/ethnicity, tumor size, stage, local treatment and year of diagnosis.

		Clear Cell RCC						
Characteristic	Before PSM				After 1:2 PSM			
	With SD, *n* = 2496 (3.0%)	Without SD, *n* = 79,650 (97.0%)	*p*-Value ^1^	|SMD| ^2^	With SD, *n* = 2496 (100%)	Without SD, *n* = 4992 (6.2%)	*p*-Value ^1^	|SMD| ^2^
**Age (years),** median (IQR)	64 (56, 71)	62 (53, 70)	**<0.001**	**0.20**	64 (56, 71)	64 (56, 71)	0.6	0.03
**Male sex**, *n*. (%)	1736 (69.6%)	50,300 (63.2%)	**<0.001**	**0.14**	1736 (69.6%)	3509 (70.3%)	0.4	0.02
**Race/ethnicity**, *n*. (%)			0.5				0.5	
Caucasian	1653 (66.2%)	52,719 (66.2%)		<0.01	1653 (66.2%)	3388 (67.9%)		0.03
Hispanic	499 (20.0%)	15,390 (19.3%)		0.02	499 (20.0%)	968 (19.4%)		0.02
African American	136 (5.4%)	4963 (6.2%)		0.03	136 (5.4%)	234 (4.7%)		0.03
Asian/Pacific Islander	166 (6.7%)	5207 (6.5%)		<0.01	166 (6.7%)	321 (6.4%)		<0.01
Other	42 (1.7%)	1371 (1.7%)		<0.01	42 (1.7%)	81 (1.6%)		0.02
**Tumor size (cm),** median (IQR)	8.5 (6.0, 11.3)	4.2 (2.8, 6.5)	**<0.001**	**0.72**	8.5 (6.0, 11.3)	8.5 (6.0, 11.4)	0.1	0.07
**Stage,** *n*. (%)			**<0.001**				0.3	
Localized	544 (21.8%)	56,823 (71.3%)		**1.20**	544 (21.8%)	1103 (22.1%)	0.9	0.01
Regional	1067 (42.7%)	16,723 (21.0%)		**0.44**	1067 (42.7%)	2141 (42.9%)		<0.01
T3-4 N0	804 (75.4%)	13,246 (79.2%)	**0.003**	**0.33**	804 (75.4%)	1620 (75.7%)	0.8	0.01
Tany N1	263 (24.6%)	3477 (20.8%)		**0.20**	263 (24.6%)	521 (24.3%)		0.01
Metastatic	885 (35.5%)	6104 (7.7%)		**0.58**	885 (35.5%)	1748 (35.0%)		0.02
**Local treatment,** *n*. (%)			**<0.001**				0.5	
Partial nephrectomy	242 (9.7%)	35,073 (44.0%)		**1.16**	242 (9.7%)	442 (8.9%)		0.03
Radical nephrectomy	2197 (88.0%)	42,091 (52.8%)		**1.08**	2197 (88.0%)	4438 (88.9%)		0.02
No local treatment *	57 (2.3%)	2486 (3.1%)		0.06	57 (2.3%)	112 (2.2%)		0.01
**Overall SMD**				**1.34**				<0.01

^1^ Wilcoxon rank-sum test; Pearson’s chi-squared test; bold values indicate reaching the significance level (set at *p* < 0.05). ^2^ Absolute standardized mean difference; bold values indicate an imbalance among groups (cut-off level set at >0.1). * Only M1 patients. Abbreviations: SD: sarcomatoid dedifferentiation.

**Table 2:** Descriptive characteristics of non-clear cell RCC patients with vs. without sarcomatoid dedifferentiation (SD), before and after 1:2 propensity score matching (PSM), relying on age, sex, race/ethnicity, tumor size, stage, histological subtype, local treatment and year of diagnosis.

	Non-Clear Cell RCC							
Characteristic	Before PSM				After 1:2 PSM			
	With SD, *n* = 501 (1.9%)	Without SD, *n* = 26,083 (98.1%)	*p*-Value ^1^	|SMD| ^2^	With SD, *n* = 501 (100%)	Without SD, *n* = 1002 (3.8%)	*p*-Value ^1^	|SMD| ^2^
**Age (years),** median (IQR)	63 (54, 71)	63 (54, 70)	0.4	0.09	63 (54, 71)	64 (55, 71)	0.2	0.04
**Male sex**, *n*. (%)	328 (65.7%)	17,972.0 (68.9%)	0.2	0.05	328 (65.7%)	684 (68.3%)	0.4	0.05
**Race/ethnicity**, *n*. (%)			0.4				0.9	
Caucasian	295 (58.9%)	15,524 (59.5%)		<0.01	295 (58.9%)	604 (60.3%)		0.03
Hispanic	67 (13.4%)	2876 (11.0%)		<0.01	67 (13.4%)	128 (12.8%)		0.02
African American	116 (23.2%)	6235 (23.9%)		0.05	116 (23.2%)	228 (22.8%)		0.09
Asian/Pacific Islander	20 (4.0%)	1130 (4.3%)		0.06	20 (4.0%)	35 (3.5%)		0.02
Other	3 (0.6%)	318 (1.2%)		0.09	3 (0.6%)	7 (0.7%)		0.01
**Tumor size (cm),** median (IQR)	8.0 (5.1, 12.0)	3.8 (2.5, 6.0)	**<0.001**	0.70	8.0 (5.1, 12.0)	8.0 (4.8, 11.4)	0.3	0.02
**Histological subtype,** *n*. (%)			**<0.001**				0.5	
Papillary	285 (56.9%)	16,224 (62.2%)		**0.45**	285 (56.9%)	611 (61.0%)		0.08
Chromophobe	152 (30.3%)	8015 (30.7%)		**0.50**	152 (30.3%)	275 (27.4%)		0.06
Collecting duct	32 (6.4%)	130 (0.5%)		**0.22**	32 (6.4%)	56 (5.6%)		0.03
Other	32 (6.4%)	1714 (6.6%)		**0.20**	32 (6.4%)	60 (6.0%)		0.02
**Stage,** *n*. (%)			**<0.001**				0.4	
Localized	193 (38.5%)	21,738 (83.3%)		**0.85**	193 (38.5%)	386 (38.5%)		<0.01
Regional	198 (39.5%)	3580 (13.7%)		**0.45**	198 (39.5%)	425 (42.4%)		0.06
T3-4 N0	120 (60.6%)	2554 (71.3%)	**0.001**	**0.27**	120 (60.6%)	252 (59.3%)	0.8	0.03
Tany N1	78 (39.4%)	1026 (28.7%)		**0.30**	78 (39.4%)	173 (40.7%)		0.05
Metastatic	110 (22.0%)	765 (2.9%)		**0.46**	110 (22.0%)	191 (19.1%)		0.07
**Local treatment,** *n*. (%)			**<0.001**				0.9	
Partial nephrectomy	84 (16.8%)	14,415 (55.3%)		**0.90**	84 (16.8%)	167 (16.7%)		<0.01
Radical nephrectomy	412 (82.2%)	11,347 (43.5%)		**0.87**	412 (82.2%)	824 (82.2%)		<0.01
No local treatment *	5 (1.0%)	321 (1.2%)		0.04	5 (1.0%)	11 (1.1%)		0.01
**Overall SMD**				**0.78**				<0.01

^1^ Wilcoxon rank-sum test; Pearson’s chi-squared test; bold values indicate reaching of significance level (set at *p* < 0.05). ^2^ Absolute standardized mean difference; bold values indicate an imbalance among groups (cut-off level set at >0.1). * Only M1 patients. Abbreviations: SD: sarcomatoid dedifferentiation.

## Data Availability

Data are publicly available within SEER database (https://seer.cancer.gov, accessed on 3 March 2026).

## Supplementary Materials

The following supporting information can be downloaded at: https://www.mdpi.com/article/10.3390/jcm15062133/s1, Table S1: Description of SEER codes used to identify patients with vs. without sarcomatoid dedifferentiation.

## Abbreviations

The following abbreviations are used in this manuscript:

SD	Sarcomatoid dedifferentiation
RCC	Renal cell carcinoma
ccRCC	Clear cell RCC
nccRCC	Non-clear cell RCC
PSM	Propensity score matching
EAPC	Estimated annual percentage changes
CSM	Cancer-specific mortality
SEER	Surveillance, Epidemiology and End Results
