# Supplementary material for: Cancer-Specific Mortality in Sarcomatoid Renal Cell Carcinoma: A Histological Subtype-Controlled Analysis
Source: J Clin Med. 2026 Mar 11;15(6):2133. doi: 10.3390/jcm15062133 (PMC13027048; doi:10.3390/jcm15062133)
Supplement: Supplementary file 1 [file jcm-15-02133-s001.zip › jcm-4158794-supplementary.pdf]

Supplementary Table S1. Description of SEER codes used to identify patients with vs. without sarcomatoid dedifferentiation.

Item	NAARC	Codes
CS-specific factor 4	#2910	00= Sarcomatoid features not present/not identified<br>010= Sarcomatoid features present/identified<br>987= Not applicable: Not a renal cell carcinoma morphology<br>988= Not applicable: Information not collected for this case<br>999= Unknown or no information/Not documented in patient record
Sarcomatoid Features Recode (2010+)	#3925	0=Sarcomatoid features not present/not identified<br>1=Sarcomatoid features present/identified<br>7=Not applicable: Not a renal cell carcinoma morphology<br>8=Not applicable: Information not collected for this case<br>9=Not documented/assessed; Unknown if assessed; No surgical resection of primary<br>14=Blank(s)
